# Supplementary material for: Composite Aramid Membranes with High Strength and pH-Response
Source: Polymers (Basel). 2021 Feb 19;13(4):621. doi: 10.3390/polym13040621 (PMC7922203; doi:10.3390/polym13040621)
Supplement: Supplementary file 1 [file polymers-13-00621-s001.pdf]

Support information

Composite Aramid Membranes with High Strength and Smart pH-response

Xiao Wang[a,e], Shi Li[a,e], Yuanyuan Tu[a,b,c,d,e], Jiwen Hu[a,b,c,d,e,*] Zhenzhu Huang[a,b,c,d,e], Shudong Lin[a,b,c,d,e], and Xuefeng Gui

[a]*Guangzhou Institute of Chemistry, Chinese Academy of Sciences, Guangzhou 510650, PR China*

[b]*Guangdong Provincial Key Laboratory of Organic Polymer Materials for Electronics, Guangzhou 510650, PR China*

[c]*CAS Engineering Laboratory for Special Fine Chemicals, Guangzhou 510650, PR China*

[d]*Incubator of Nanxiong CAS Co., Ltd., Nanxiong 512400, PR China*

[e]*University of Chinese Academy of Sciences, Beijing 100049, PR China*

*Corresponding author: Prof. Hu,
E-mail: hjw@gic.ac.cn

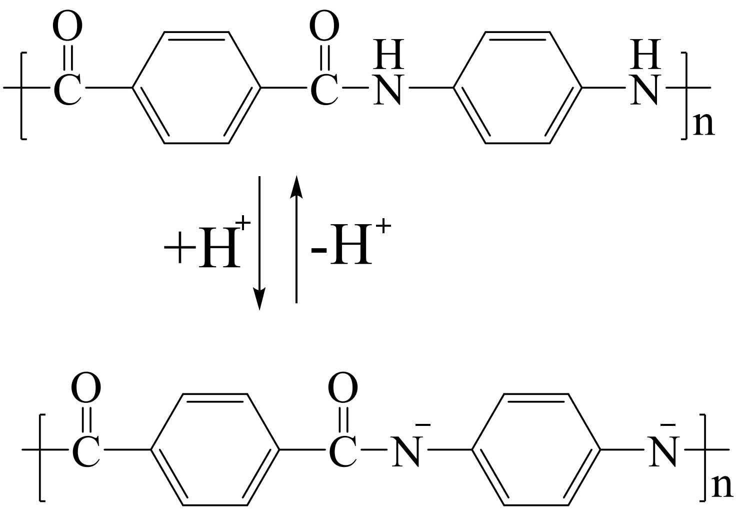

Figure S1 The deprotonation and protonation of PPTA

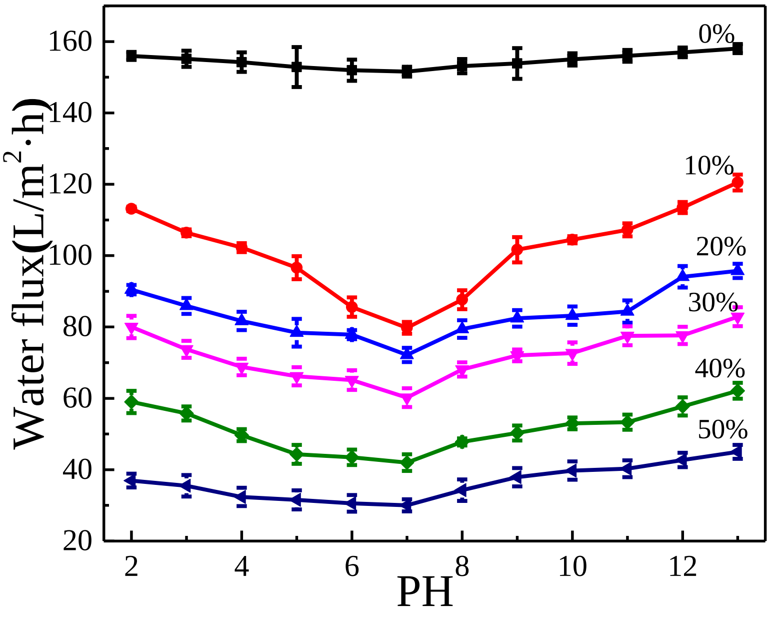

Figure S2 Trans-membrane fluxes of aqueous solutions at different pH values across ANF/HANF membranes with different contents of HANF

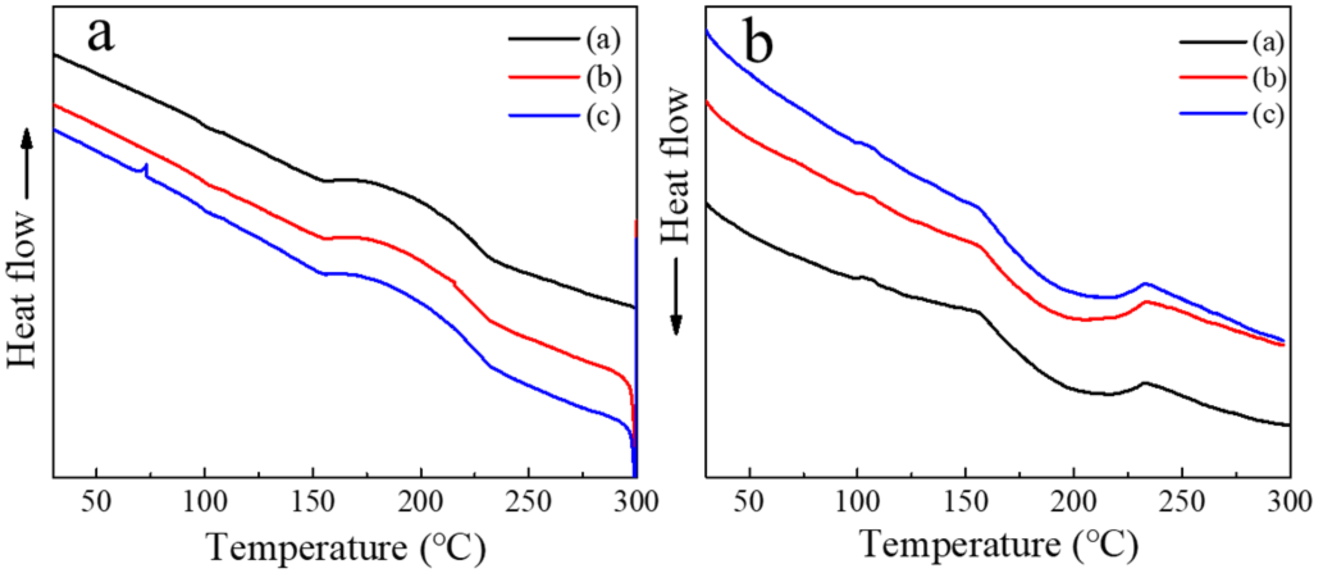

Figure S3 The DSC curve of cooling process(a) and heating process(b) ((a): ANF membrane, (b): ANF/HANF membrane, (c) HANF)

From the DSC cure, we could see that the Tg of the ANF membrane, ANF/HANF membrane and HANF was 158.02 ºC, 157.75 ºC, 157.17 ºC respectively. This result showed that the acidification of ANF to HANF had little influence on the Tg, and the Tg of the film prepared by the mixture of the two was between that of ANF and HANF. The reason might be that in the acidizing process, the amide bond on the surface of ANF fiber was broken to form HANF with ammonium and carboxyl groups, and the main internal structure of ANF did not change.

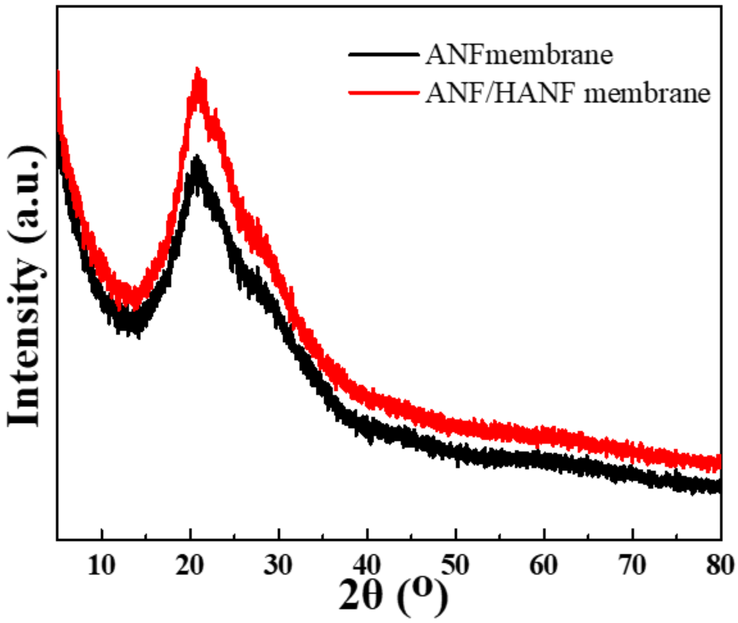

Figure S4 XRD patterns of the ANF membranes and ANF/HANF composite membranes (HANF: 50wt% of ANF)

The X-ray diffraction (XRD) diffraction analysis was performed with a Bruker D8 advance XRD diffractometer using Cu Kα radiation (λ = 0.154 06 nm, 40 kV and 30 mA). From the XRD spectra, we could see that the XRD patterns of the ANF/HANF composite membranes was similar to that of the ANF membranes. We could see that there was a diffuse hump symbolizing an indefinite form at about 20°, which indicated that the addition of HANFs has no effect on the crystallinity of ANF membrane, and the ANF and ANF/HANF films were all amorphous.

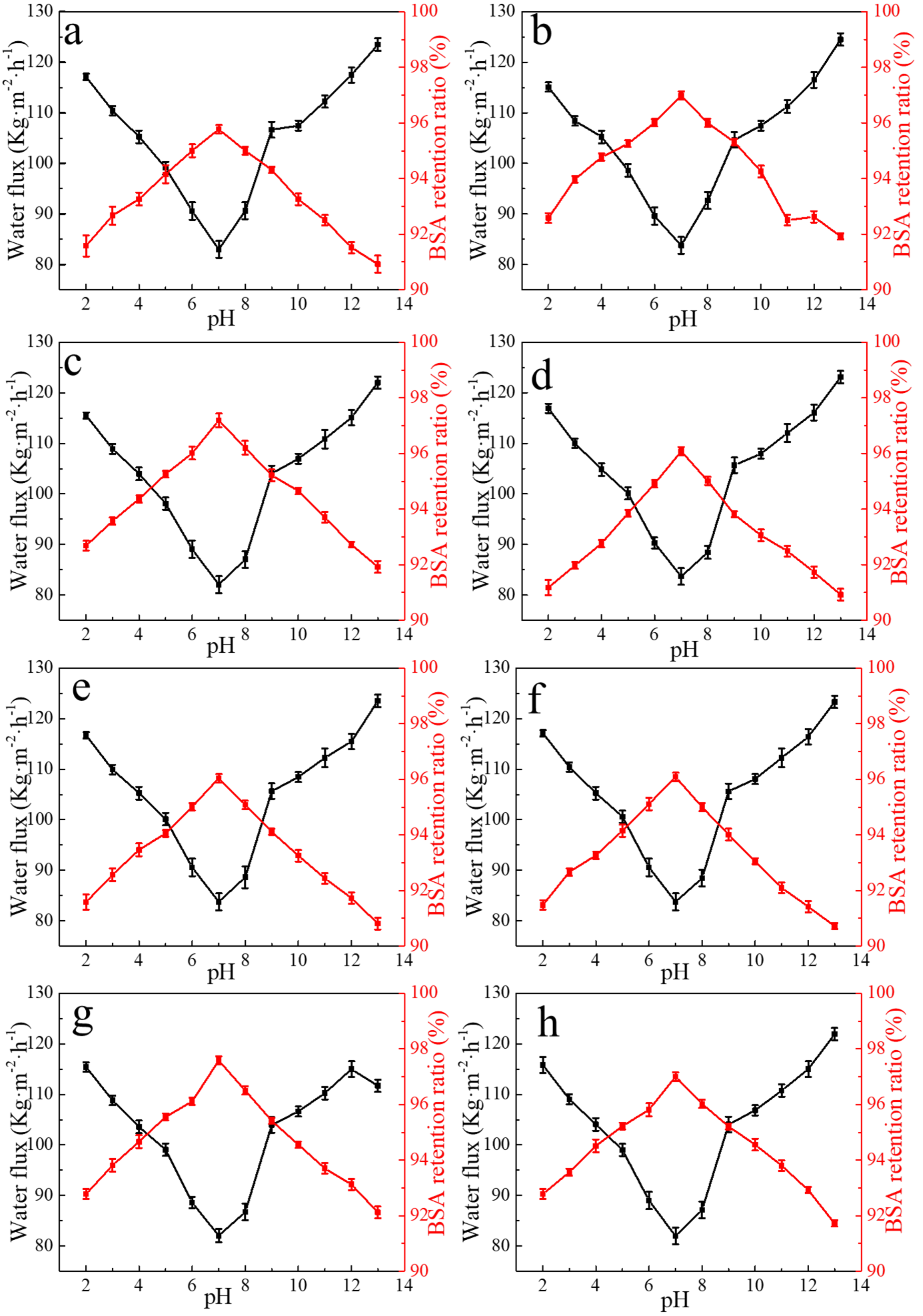

Figure S5. The variation of water flux (black line) and BSA retention ratio (red line) of the membranes after soaked in different solvet (a: DMSO; b: NMP, c: DMF, d: DMAC, e: THF, f: DMC, g: PhMe, h: MeOH) for 15 days with pH value.

Futher to the chemical stability test, the variation of water flux and BSA retention ratio of the membranes after soaked in different solvent for 15 days with pH value were also characterized. From figure S5, we could see that the trend of the variation of water flux and BSA retention ratio of the membranes with pH value remained unchanged, and the value of the flux and BSA retention of the membranes have a little change. The value of the water flux of membranes increased slightly in the solvent of DMSO, DMAC, THF, DMC, and decreased slightly in the solvent of NMP, DMF, PhMe, MeOH. At the same time the change of BSA retention ratio was just opposite to that of water flux. While the value the amount of change was very slight. Therefore, the membranes prepared by this method had good chemical stability.
